# Biosynthetic origin of butyrolactol A, an antifungal polyketide produced by a marine-derived *Streptomyces*

**DOI:** 10.3762/bjoc.13.47

**Published:** 2017-03-08

**Authors:** Enjuro Harunari, Hisayuki Komaki, Yasuhiro Igarashi

**Affiliations:** 1 Biotechnology Research Center and Department of Biotechnology, Toyama Prefectural University, 5180 Kurokawa, Imizu, Toyama 939-0398, Japan; 2 Biological Resource Center, National Institute of Technology and Evaluation (NBRC), 2-5-8 Kazusakamatari, Kisarazu, Chiba 292-0818, Japan

**Keywords:** biosynthesis, butyrolactol, contiguous polyol, hydroxymalonyl-ACP, polyketide, *Streptomyces*, *tert*-butyl

## Abstract

Butyrolactol A is an antifungal polyketide of *Streptomyces* bearing an uncommon *tert*-butyl starter unit and a polyol system in which eight hydroxy/acyloxy carbons are contiguously connected. Except for its congener butyrolactol B, there exist no structurally related natural products to date. In this study, inspired by our previous genomic analysis, incorporation of ^13^C- and ^2^H-labeled precursors into butyrolactol A was investigated. Based on the labeling pattern and sequencing analytical data, we confirmed that the *tert*-butyl group is derived from valine and its *C*-methylation with methionine and the polyol carbons are derived from a glycolysis intermediate, possibly hydroxymalonyl-ACP.

## Introduction

Actinomycetes produce structurally diverse secondary metabolites with pharmaceutically useful bioactivities. Importantly, members of the genus *Streptomyces* have been the main source of drug discovery programs due to their high capacity in secondary metabolism including polyketides, peptides, terpenoids, alkaloids, and amino acid/carbohydrate/nucleic acid derivatives [1–2]. One of the largest groups of bacterial secondary metabolites is polyketide from which a range of clinically used drugs have been developed. Polyketides still remain in the focus of drug development because of their structural complexity that can provide attractive templates for new pharmacophores [3]. While the frequency of discovering new skeletons from actinomycetes seems declining, biosynthetic analysis of structurally unique known compounds and the following bioengineering of biosynthetic genes are currently becoming an essential part of the creation of new drug-like structures [4–8].

**Figure 1:**
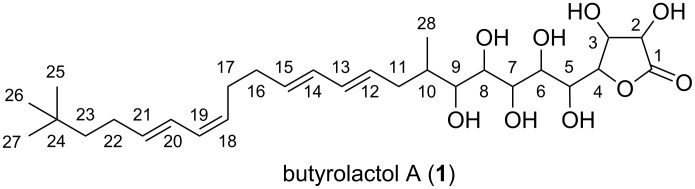
The structure of butyrolactol A (**1**).

Butyrolactol A (**1**) is an antifungal polyketide first isolated from *Streptomyces rochei* S785-16 [9] (Figure 1). The left half of **1** is the hydrophobic unconjugated tetraene system including one *Z*-olefin with a terminal *tert*-butyl group, whereas the hydrophilic polyol system bearing a γ-lactone terminus constitutes the right half of the molecule. To date, no structurally related natural products are known except for its demethyl congener butyrolactol B that was also isolated from the same strain and has an isopropyl group instead of the *tert*-butyl terminus [9]. Very recently, isolation of butyrolactols C and D was presented but the details are not available in public domains [10]. **1** has a broad antimicrobial activity against fungi ranging from *Candida albicans* to *Trichophyton mentagrophytes* with comparative activity to nystatin [9]. Despite the uniqueness of the structure and the antifungal potency, no further research has been reported for **1**.

**Figure 2:**
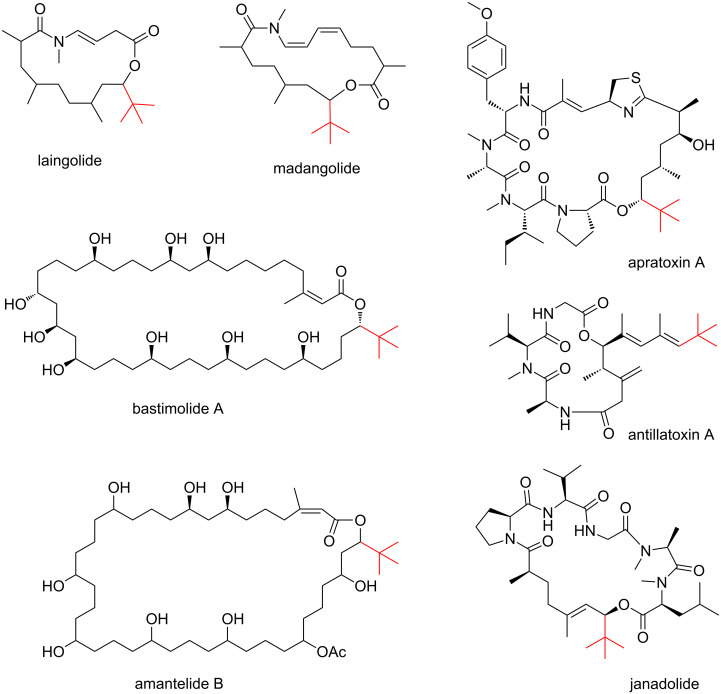
Cyanobacterial polyketides bearing a *tert*-butyl group.

**Figure 3:**
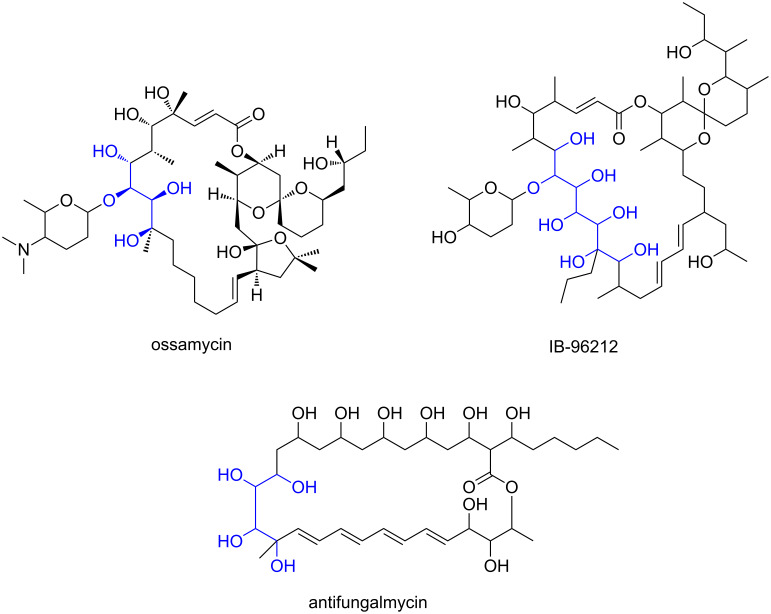
Actinomycete metabolites possessing a contiguous 1,2-diol system.

There are two interesting aspects in the structure of butyrolactol A (**1**). First, among the polyketides, a *tert*-butyl group has been found exclusively in metabolites of marine cyanobacteria except for **1** [11–14] (Figure 2). Although no experimental evidence is available, pivaloyl-CoA (2,2-dimethylpropanoyl-CoA) is supposed to be a starter for its biosynthesis [15]. Additionally, trimethylation of malonyl-CoA is proposed for the synthesis of the *tert*-butyl starter in the biosynthesis of apratoxin A [16]. Another intriguing feature of this molecule is the highly oxygenated carbon chain in which eight hydroxy groups, one of which is used for lactone formation, are contiguously aligned. A 1,3-diol is a common structural element in aliphatic polyketides because the incorporation of malonate-precursors gives rise to the alternative alignment of the methylene and the oxygenated carbons. Meanwhile, a 1,2-diol in polyketides is known to be formed by hydroxylation of methylene carbons as seen in the biosynthesis of erythromycin or amphotericin B [17–18]. The contiguously hydroxylated carbon chain of **1** is quite unusual as a polyketide. Examples of similar but shorter polyol carbon chains are ossamycin [19], IB-96212 [20], and antifungalmycin [21], all of which are the secondary metabolites of actinomycetes (Figure 3).

In our investigation on secondary metabolites of marine actinomycetes, butyrolactol A (**1**) was found to be produced by a *Streptomyces* strain collected from deep sea water of the Toyama Bay, Japan. In order to get insight into the construction of the above-mentioned unusual structures, we performed an in silico analysis of the biosynthetic genes of **1** through draft genome sequencing and proposed its biosynthetic pathway [22]. In this study, biosynthetic precursors of **1** were investigated for further genetic and enzymatic studies.

## Results and Discussion

**Figure 4:**
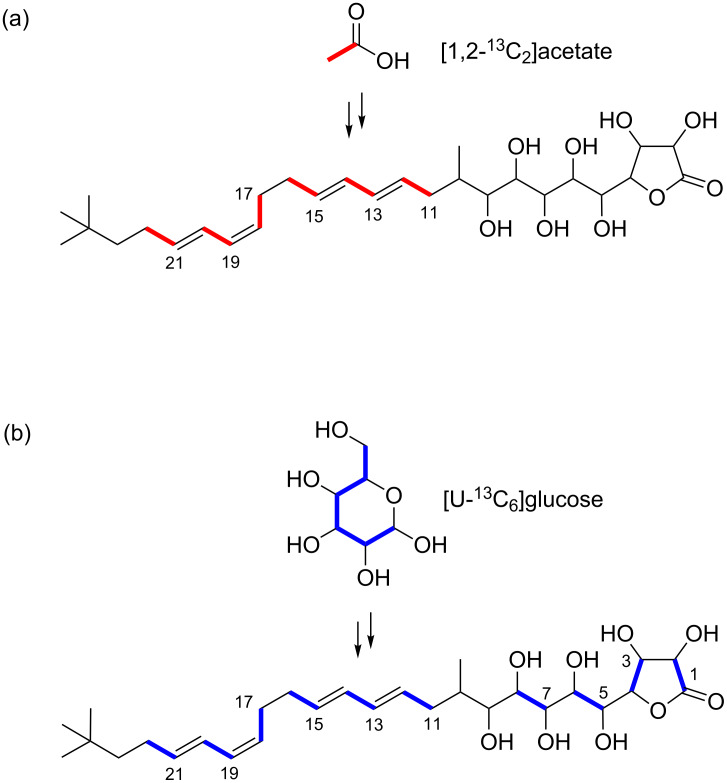
Feeding experiments of ^13^C-labeled precursors into **1** detected by 2D-INADEQUATE NMR experiments. (a) [1,2-^13^C_2_]acetate; (b) [U-^13^C_6_]glucose.

It was obvious from its structure that **1** was synthesized through the malonate pathway. First, [1,2-^13^C_2_]acetate was fed to the culture to ensure the alignment of malonate units. In the ^13^C NMR spectrum, split signals arising from ^13^C–^13^C couplings were observed for six pairs of carbons: C-11/C-12, C-13/C-14, C-15/C-16, C-17/C-18, C-19/C-20, and C-21/C-22 (Table 1, Figure S1 in Supporting Information File 1). In the 2D-INADEQUATE spectrum of ^13^C-labeled **1** obtained with a parameter set optimized for ^1^*J*_CC_ 50 Hz, cross peaks derived from the intact ^13^C_2_ acetate units were detected for the carbon pairs mentioned above (Table 1, Figure 4a).

**Table 1:** Incorporation of ^13^C-labeled precursors into **1**.

Position	δ_C_	[1,2-^13^C_2_]acetate			[U-^13^C_6_]glucose			[1-^13^C]propionate	L-[*methyl*-^13^C]methionine
				
		^1^*J*_CC_ (Hz)	2D-INADEQUATE		^1^*J*_CC_ (Hz)	2D-INADEQUATE		relative enrichments^a^	

1	175.3				56	2		0.9	0.9
2	74.7				56	1		1.0	1.0
3	72.9				39	4		0.9	1.0
4	80.0				39	3		1.0	1.1
5	66.9				44	6		1.0	1.0
6	68.9				44	5		0.9	0.9
7	68.9				45	8		1.0	1.1
8	69.7				45	7		1.0	1.1
9	73.1							**4.8**	1.2
10	36.2							1.1	1.1
11	36.6	41	12		40	12		1.2	1.2
12	131.9	41	11		40	11		1.3	1.2
13	131.9	55	14		57	14		1.3	1.2
14	131.3	55	13		57	13		1.2	1.0
15	131.3	43	16		43	16		1.2	1.0
16	32.6	43	15		43	15		1.2	1.3
17	27.5	42	18		43	18		1.4	1.3
18	129.2	42	17		43	17		1.2	1.0
19	129.6	55	20		55	20		1.4	1.1
20	125.7	55	19		55	19		1.3	1.1
21	135.9	43	22		43	22		1.3	1.0
22	28.1	43	21		43	21		1.2	1.1
23	43.7							1.6	1.2
24	30.6							1.2	1.0
25	29.7							0.9	**7.1**
26	29.7							0.9	**7.1**
27	29.7							0.9	**7.1**
28	16.2							1.1	1.2

^a^The ^13^C signal intensity of each peak in labeled **1** divided by that of the corresponding signal in unlabeled **1**, respectively, normalized to give an enrichment ratio of **1** for the unenriched C-2 peak.

**Figure 5:**
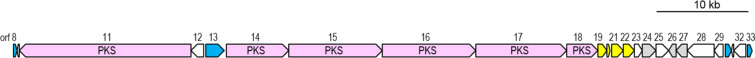
Organization of the biosynthesis gene cluster for **1**. Blue, transcriptional regulator; pink, PKS for polyketide backbone of **1**; yellow, genes for biosynthesis of hydroxymalonyl-ACP; gray, transporter.

**Figure 6:**
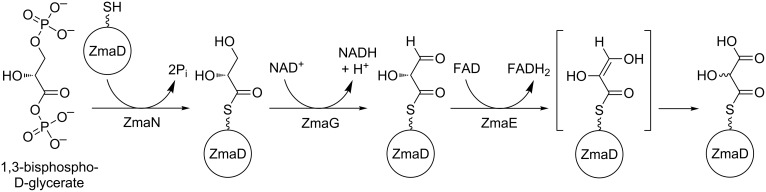
Biosynthetic pathway of hydroxymalonyl-ACP. Adapted from [24].

According to the incorporation result of the doubly labeled acetate, malonyl-CoA is not the extender unit for the lactone (C-1 to C-4) and the pentaol (C-5 to C-9) moieties (Figure 3), suggesting that the contiguous polyol system is not formed by methylene hydroxylation. Another possible pathway for 1,2-diol formation is the incorporation of hydroxymalonyl-ACP from a glycolytic intermediate for chain elongation [23]. To investigate this possibility, we conducted a feeding experiment of [U-^13^C_6_]glucose which could label carbons derived from malonyl-CoA and hydroxymalonyl-ACP. In the ^13^C NMR spectrum, ^13^C–^13^C couplings were observed for C-1/C-2, C-3/C-4, C-5/C-6, C-7/C-8 in addition to the carbon pairs C-11/C-12, C-13/C-14, C-15/C-16, C-17/C-18, C-19/C-20, and C-21/C-22 (Table 1, Figure S3 in Supporting Information File 1). The 2D-INADEQUATE spectrum showed cross peaks for the above-mentioned two-carbon units derived from the glycolytic degradation of [U-^13^C_6_]glucose (Table 1, Figure 4b). Combined with the acetate-labeling result, this labeling pattern suggested that the carbons from C-1 to C-8 are derived from hydroxymalonyl-ACP. This conclusion is supported by the sequencing analysis of the gene cluster for butyrolactol biosynthesis (Figure 5, Table 2). Four genes coding homologues of enzymes involved in hydroxymalonyl-ACP formation in the zwittermicin biosynthesis (ZmaN, ZmaD, ZmaG, and ZmaE) (Figure 6) [24] are present in the downstream of the butyrolactol PKS cluster. Genes coding for *O*-methyltransferase homologues responsible for *O*-methylation of hydroxymalonyl-ACP were not found near the cluster. Hydroxymalonyl-ACP was first identified as an unusual polyketide extender for zittermicin from *Bacillus cereus* [25]. The occurrence of this uncommon extender unit is limited to some bacterial species *Bacillus* [25–26], *Xenorhabdus* [27], *Paenibacillus* [28], and *Streptomyces* [29–30].

**Table 2:** Annotated putative ORFs in biosynthetic gene cluster and neighboring genes of **1**.

Orf10-	Accession no.	Size (aa)	Proposed function	BLAST search	

				Protein homolog, Origin, Accession number	%^a^

8	WP_055469543	127	HxlR family transcriptional regulator	HxlR family transcriptional regulator, *Streptomyces* sp. NRRL F-7442, KOX41174	99/100
10^b^	WP_030405160	71	acetyl-CoA carboxylase biotin carboxyl carrier protein subunit	acetyl-CoA carboxylase, *Streptomyces* sp. NRRL F-7442, KOX41173	100/100
11^b^	WP_055469545	6,065	PKS	FscE, *Streptomyces cattleya*, AEW99638	76/82
12^b^	WP_055469546	473	propionyl-CoA carboxylase subunit beta	propionyl-CoA carboxylase subunit beta, *Streptomyces* sp. NRRL F-7442, KOX41172	99/99
13	WP_055469547	676	helix-turn-helix transcriptional regulator	regulator, *Streptomyces* sp. NRRL F-7442, KOX41171	99/99
14	WP_055469666	2,075	PKS	polyketide synthase type I, *Streptomyces cattleya*, AEW99622	71/80
15	WP_055469548	3,365	PKS	FscC, *Streptomyces cattleya*, AEW99623	71/79
16	WP_055469549	3,462	PKS	polyketide synthase (fragment), *Streptomyces cattleya*, KOX46585	99/99
17	WP_055469550	3,135	PKS	polyketide synthase, *Streptomyces* sp. NRRL F-7442, KOX46586	99/99
18	WP_055469551	1,169	PKS	short-chain dehydrogenase, *Streptomyces* sp. NRRL-7442, KOX46587	99/99
19	WP_055469552	301	3-hydroxyacyl-CoA dehydratase	3-hydroxybutyryl-CoA dehydrogenase, *Streptomyces* sp. NRRL F-7442, KOX46623	98/99
20	WP_030403675	88	ACP	Acyl carrier protein, *Streptomyces* sp. NRRL F-7442, KOX46588	100/100
21	WP_055469553	381	acyl-CoA dehydratase	acyl-CoA dehydrogenase, *Streptomyces* sp. NRRL F-7442, KOX46589	99/99
22	WP_055469554	356	glyceroyl-ACP biosynthesis protein	FkbH, *Streptomyces* sp. NRRL F-7442, KOX46590	99/99
23	WP_030403672	261	thioesterase	thioesterase, *Streptomyces* sp. NRRL F-7442, KOX46591	100/100
24	WP_055469555	448	MFS transporter	major facilitator superfamily permease, *Streptomyces cattleya*, AEW99632	78/84
25	WP_055469556	408	hypothetical protein	uncharacterized protein, *Streptomyces* sp. NRRL F-7442, KOX46624	99/100
26^b^	WP_055469557	253	multidrug ABC transporter permease	multidrug ABC transporter permease, *Streptomyces* sp. NRRL F-7442, KOX46592	99/99
27^b^	not registered in GenBank	373	ABC transporter ATP-binding protein	ABC transporter related protein, *Streptomyces cattleya*, AEW99635	82/89
28^b^	WP_055469558	868	hypothetical protein	beta-ketoacyl synthase, *Streptomyces* sp. NRRL F-7442, KOX46593	99/99
29^b^	WP_055469559	290	ketopantoate reductase	ketopantoate reductase, *Streptomyces* sp. NRRL F-7442, KOX46594	98/99
30	WP_055469560	209	TetR family transcriptional regulator	TetR family transcriptional regulator, *Streptomyces* sp. NRRL F-7442, KOX46595	99/99
31	WP_059296555	65	chitinase	secreted chitinase, *Streptomyces coelicolor*, NP_733504	83/87
32^b^	WP_055469561	407	FAD-dependent oxidoreductase	FAD-dependent oxidoreductase, *Streptomyces* sp. NRRL F-7442, KOX46596	98/99
33	WP_030403663	168	MarR family transcriptional regulator	MarR family transcriptional regulator, Streptomyces sp. NRRL F-7442, KOX46597.1	100/100

^a^Identity/similarity; ^b^encoded in complementary strand.

**Figure 7:**
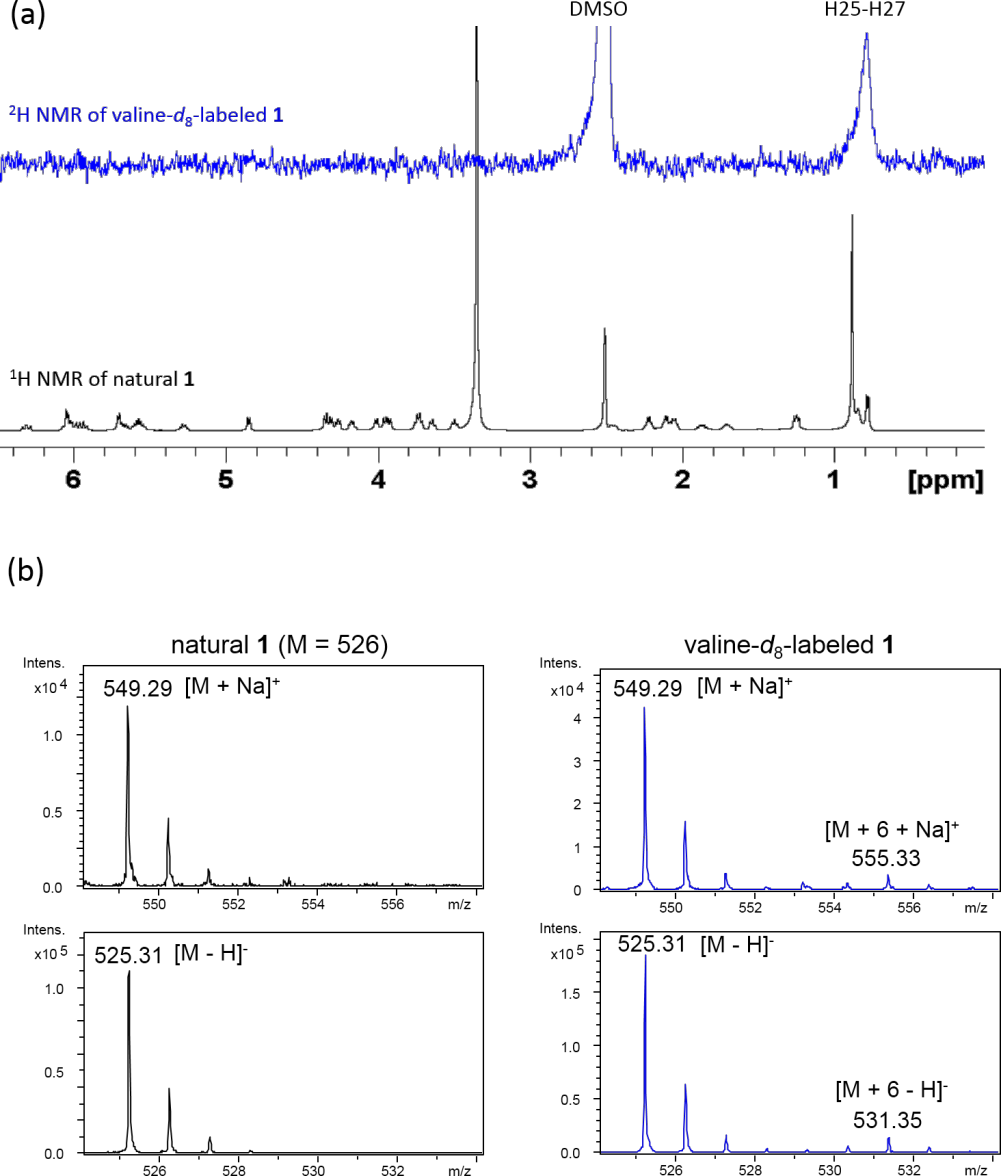
Incorporation of L-valine-*d*_8_ into **1**. (a) ^1^H NMR spectrum of natural **1** and ^2^H NMR spectrum of L-valine-*d*_8_-labeled **1**. (b) ESIMS spectra of natural **1** and L-valine-*d*_8_-labeled **1**.

Methylmalonyl-CoA was readily predicted as the origin of the methyl-branched three-carbon fragment (C-9/C-10/C-28). Actually, intense enhancement of the C-9 carbon signal was observed by feeding of [1-^13^C]propionate (Table 1). The remaining carbons not labeled by malonate-type precursors were the terminal *tert*-butyl carbons (C-23 to C-27). The origin of the *tert*-butyl group in polyketide biosynthesis is still unknown, however, the *tert*-butyl functionality of bottromycin and polytheonamide was shown to be produced by radical *C*-methylation of the isopropyl group of valine [31–32]. By analogy, the *tert*-butyl portion of **1** was most likely supplied through the *C*-methylation of valine. To examine this possibility, feeding experiments of L-[*methyl*-^13^C]methionine and L-valine-*d*_8_ were carried out. As expected, the *tert*-butylmethyl carbons (C-25, C-26, C-27) were labeled as a result of L-[*methyl*-^13^C]methionine incorporation (Table 1). In addition, the ^2^H (deuterium) NMR spectrum showed deuterium signals for the methyl group (H-25, H-26, H-27) of L-valine-*d*_8_-labeled **1** (Figure 7a). The mass spectrum of the L-valine-*d*_8_-labeled **1** displayed the molecular ion with a mass increment of 6 Da (Figure 7b), corresponding to the incorporation of six deuterium atoms into the terminal methyl groups. Based on these results from precursor-feeding experiments, we concluded that the *tert*-butyl group and the adjacent methylene carbon (C-23) are derived from valine and the *S*-methyl carbon of methionine. It is controversial whether pivaloyl CoA is loaded onto the ACP as a starter or isobutyl-CoA is used as a starter and *C*-methylation takes place afterwards. The signature sequence region of the acyltransferase domain of the PKS starter loading module for butyrolactol biosynthesis (FAGHS) shares some amino acid residues with the known loading module of isobutyl CoA (bafilomycin: LAAHS [33], α-lipomycin: LAAHS [34], tautomycin: LAAHS [35]). Meanwhile, it is known that the substrate recognition is not strict for the loading module of avermectin (VPAHS) [36] and myxalamide (VAVHS) [37] which accept both isobutyl-CoA and 2-methylbutyl-CoA. In addition, genes coding for *C*-methyltransferase are not present near the butyrolactol PKS genes. Further enzymatic studies are necessary to establish the order of the starter loading/*C*-methylation events.

## Conclusion

**Figure 8:**
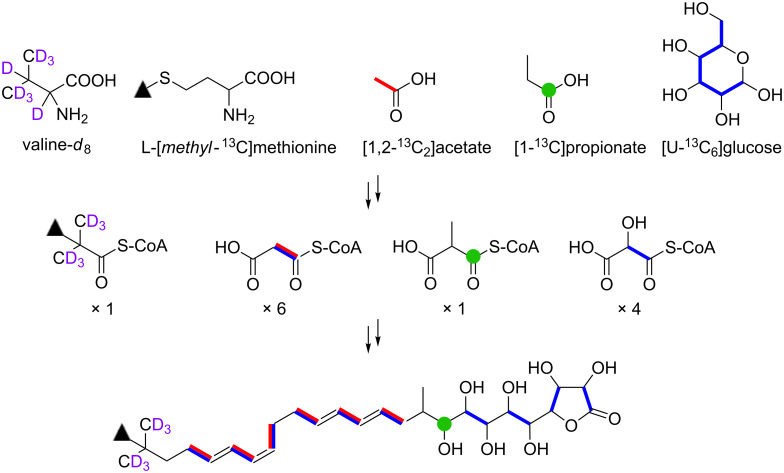
Incorporation of ^13^C- and ^2^H-labeled precursors into **1**.

In summary, we elucidated the biosynthetic origin of butyrolactol A (**1**) on the basis of the feeding experiments of isotope-labeled precursors in combination with the bioinformatics analysis of its biosynthetic genes. The overall result of labeling experiments is summarized in Figure 8. The *tert*-butyl group was shown to be derived from the *C*-methylated isopropyl group of valine. This is the first study that experimentally identified the precursor of a *tert*-butyl group in a polyketide backbone. The unusual contiguous polyol system comprising eight hydroxylated carbons was proved to be arising from the chain extension using hydroxymalonyl-ACP by labeling experiments of [1,2-^13^C_2_]acetate and [U-^13^C_6_]glucose. This conclusion is consistent with our previous bioinformatic prediction that suggested the presence of genes necessary for the supply of hydroxymalonyl-ACP adjacent to the PKS gene cluster of the butyrolactol biosynthesis. The results obtained in this study provide useful information for further biosynthetic studies and genome mining of structurally unique/novel secondary metabolites.

## Experimental

### General experimental procedures

Sodium [1,2-^13^C_2_]acetate and L-valine-*d*_8_ were purchased from Cambridge Isotope Laboratories, Inc. [U-^13^C_6_]Glucose, sodium [1-^13^C]propionate, and L-[*methyl*-^13^C]methionine were purchased from Sigma-Aldrich Co. LLC. ^1^H and ^13^C NMR spectra were obtained on a Bruker AVANCE 500 spectrometer in DMSO-*d*_6_ using the signal of the residual solvent signals (δ_H_ 2.50, δ_C_ 40.0) as an internal standard. The ^2^H NMR spectrum was obtained on a Bruker AVANCE 500 spectrometer in DMSO. Chemical shifts were referenced to the solvent signal (δ_H(D)_ 2.50). ESITOFMS were recorded on a Bruker microTOF focus.

### Microorganism

*Streptomyces* sp. strain TP-A0882 was isolated from a deep seawater collected in the Toyama Bay, Japan. The strain was identified as a member of the genus *Streptomyces* on the basis of 99.9% 16S rRNA gene sequence identity (1533 nucleotides; NCBI GneBank number BBOK01000029.1) with *Streptomyces diastaticus* subsp*. ardesiacus* NRRL B-1773^T^ (accession number DQ026631).

### Fermentation

Strain TP-A0882 growing on a plate culture was inoculated into a 500 mL K-1 flask containing 100 mL of the V-22 seed medium consisting of soluble starch 1.0%, glucose 0.5%, NZ-case (Wako Pure Chemical Industries, Ltd.) 0.3%, yeast extract (Kyokuto Pharmaceutical Industrial Co., Ltd.), 0.2%, Tryptone (Difco Laboratories) 0.5%, K_2_HPO_4_ 0.1%, MgSO_4_·7H_2_O 0.05%, and CaCO_3_ 0.3% (pH 7.0). The flask was placed on a rotary shaker (200 rpm) at 30 °C for 4 days. Then, the seed culture (3 mL) was transferred into 500 mL K-1 flasks each containing 100 mL of the A-3M production medium consisting of soluble starch 2.0%, glycerol 2.0%, glucose 0.5%, Pharmamedia (Traders Protein) 1.5%, yeast extract 0.3%, and Diaion HP-20 resin (Mitsubishi Chemical Corporation) 1%. The pH of the medium was adjusted to 7.0 before sterilization. The inoculated flasks were placed on a rotary shaker (200 rpm) at 30 °C for 6 days.

### Extraction and isolation

After incubation, 100 mL of 1-butanol was added to each flask, and the flasks were allowed to shake for an hour. The mixture was centrifuged at 6,000 rpm for 10 min and the organic layer was collected from the aqueous layer. The solvent was removed by evaporation to give 1.6 g of a crude extract from 1 L of culture. This crude extract was fractionated using silica gel column chromatography with a step gradient of CHCl_3_–MeOH (1:0, 20:1, 10:1, 4:1, 2:1, 1:1, and 0:1 v/v). Fraction 4 (4:1) containing **1** was concentrated to give 16.2 mg of dark yellow gum. The final purification was achieved by preparative HPLC (Cosmosil 5C18-AR-II, 10 × 250 mm, 4 mL/min) using a gradient of MeCN/0.1% HCO_2_H (MeCN concentration: 50–100% for 0–30 min) at 4 mL/min, yielding **1** (2.7 mg) with a retention time of 26.7 min.

### Incorporation of ^13^C- and ^2^H-labeled precursors

Feeding experiments were performed for sodium [1,2-^13^C_2_]acetate, [U-^13^C_6_]glucose, sodium [1-^13^C]propionate, L-[*methyl*-^13^C]methionine, and L-valine-*d*_8_. Inoculation, cultivation, and purification were performed in the same manner as described above. Addition of ^13^C- and ^2^H-labeled precursors was initiated at 48 h after inoculation and periodically carried out every 24 h for four times. After further incubation for 24 h, the cultures were extracted with 1-butanol.

1. Sodium [1,2-^13^C_2_]acetate: After feeding of sodium [1,2-^13^C_2_]acetate (total 800 mg; 20 mg × 10 flasks × 4 days), 3.6 mg of ^13^C-labeled **1** was obtained from 1 L of culture.
2. [U-^13^C_6_]Glucose: After feeding of [U-^13^C_6_]glucose (total 800 mg; 20 mg × 10 flasks × 4 days), 2.5 mg of ^13^C-labeled **1** was obtained from 1 L of culture.
3. Sodium [1-^13^C]propionate: After feeding of sodium [1-^13^C]propionate (total 800 mg; 20 mg × 10 flasks × 4 days), 2.1 mg of ^13^C-labeled **1** was obtained from 1 L of culture.
4. L-[*Methyl*-^13^C]methionine: After feeding of L-[*methyl*-^13^C]methionine (total 80 mg; 2.0 mg × 10 flasks × 4 days), 3.2 mg of ^13^C-labeled **1** was obtained from 1 L of culture.
5. L-Valine-*d*_8_: After feeding of L-valine-*d*_8_ (total 80 mg; 2.0 mg × 10 flasks × 4 days), 2.1 mg of deuterated **1** was obtained from 1 L of culture.

## Supporting Information

File 1 NMR spectra of ^13^C- and ^2^H-labeled **1**.
