# Supplementary material for: Biosynthetic origin of butyrolactol A, an antifungal polyketide produced by a marine-derived Streptomyces
Source: Beilstein J Org Chem. 2017 Mar 8;13:441–50. doi: 10.3762/bjoc.13.47 (PMC5355916; doi:10.3762/bjoc.13.47)

Supporting Information

for

Biosynthetic origin of butyrolactol A, an antifungal polyketide produced by a marine-derived *Streptomyces*

Enjuro Harunari[1], Hisayuki Komaki[2] and Yasuhiro Igarashi[*1]

Address: [1]Biotechnology Research Center and Department of Biotechnology, Toyama Prefectural University, 5180 Kurokawa, Imizu, Toyama 939-0398, Japan and [2]Biological Resource Center, National Institute of Technology and Evaluation (NBRC), 2-5-8 Kazusakamatari, Kisarazu, Chiba 292-0818, Japan

E-mail: Yasuhiro Igarashi[*] - yas@pu-toyama.ac.jp
[*]Corresponding author

NMR spectra of ^{13}C- and ^{2}H-labeled 1

Table of Contents

Figure S1. ^{13}C NMR spectrum of butyrolactol A (**1**) labeled with [1,2-$^{13}C_2$]acetate (125 MHz, DMSO-d_6)
Figure S2. 2D-INADEQUATE spectrum of **1** labeled with [1,2-$^{13}C_2$]acetate (125 MHz, DMSO-d_6)
Figure S3. ^{13}C NMR spectrum of **1** labeled with [U-$^{13}C_6$]glucose (125 MHz, DMSO-d_6)
Figure S4. 2D-INADEQUATE spectrum of **1** labeled with [U-$^{13}C_6$]glucose (125 MHz, DMSO-d_6)
Figure S5. ^{13}C NMR spectrum of **1** labeled with [1-^{13}C]propionate (125 MHz, DMSO-d_6)
Figure S6. ^{13}C NMR spectrum of **1** labeled with L-[*methyl*-^{13}C]methionine (125 MHz, DMSO-d_6)
Figure S7. ^{1}H NMR spectrum of **1** (500 MHz, DMSO-d_6)
Figure S8. HRESITOFMS spectrum of **1**

Figure S1: ^{13}C NMR spectrum of butyrolactol A (**1**) labeled with [1,2-$^{13}C_2$]acetate (125 MHz, DMSO-d_6)

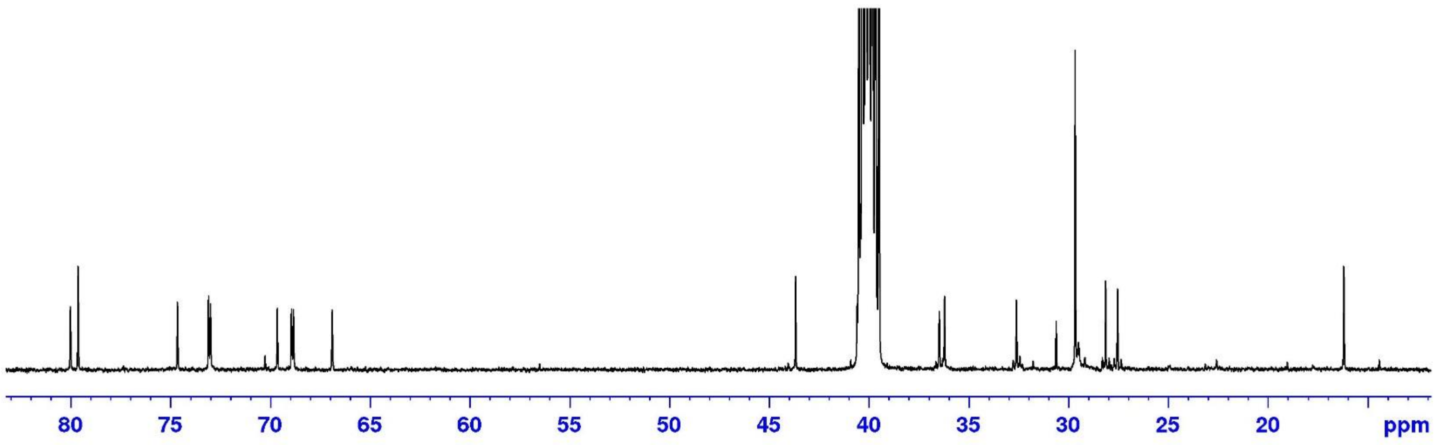

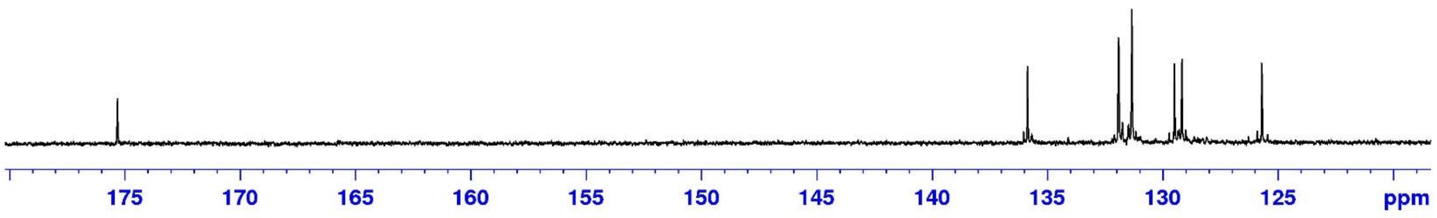

Figure S2: 2D-INADEQUATE spectrum of **1** labeled with [1,2-$^{13}C_2$]acetate (125 MHz, DMSO-d_6)

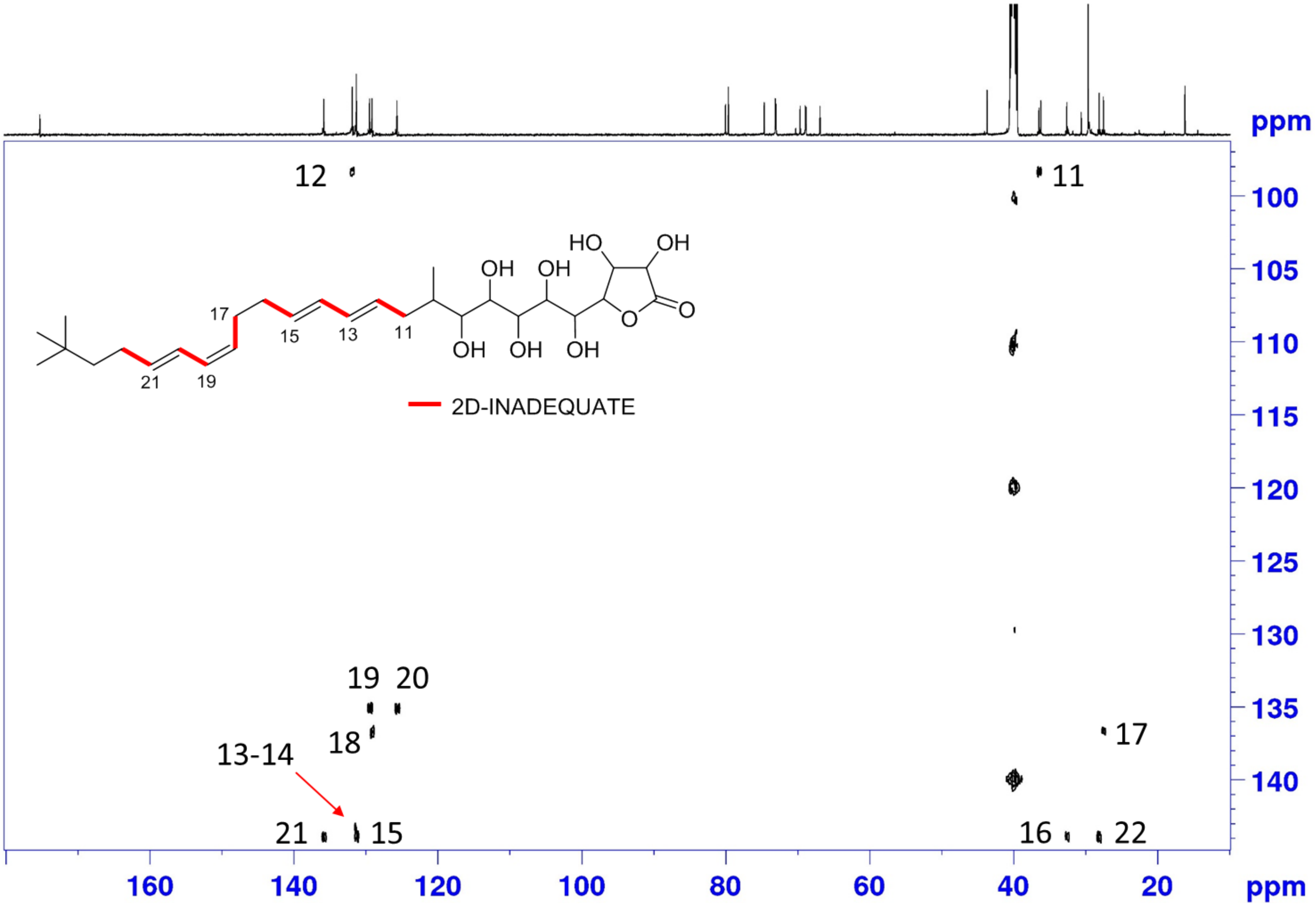

Figure S3: ^{13}C NMR spectrum of **1** labeled with [U-$^{13}C_6$]glucose (125 MHz, DMSO-d_6)

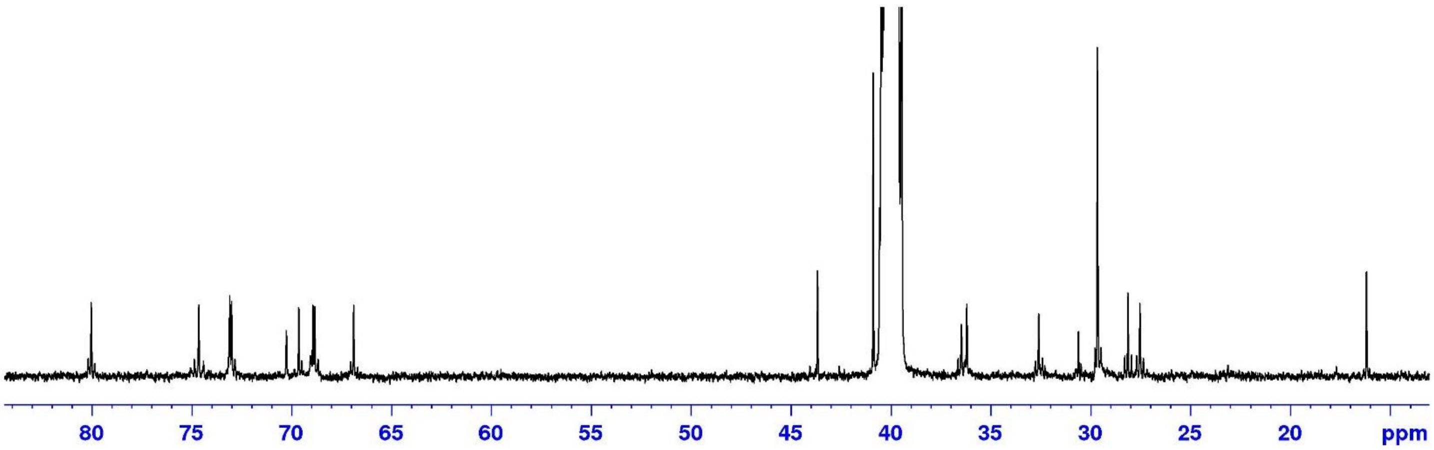

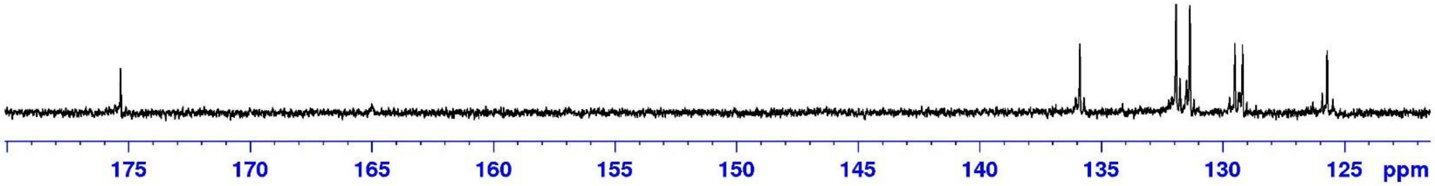

Figure S4: 2D-INADEQUATE spectrum of **1** labeled with [U-$^{13}C_6$]glucose (125 MHz, DMSO-d_6)

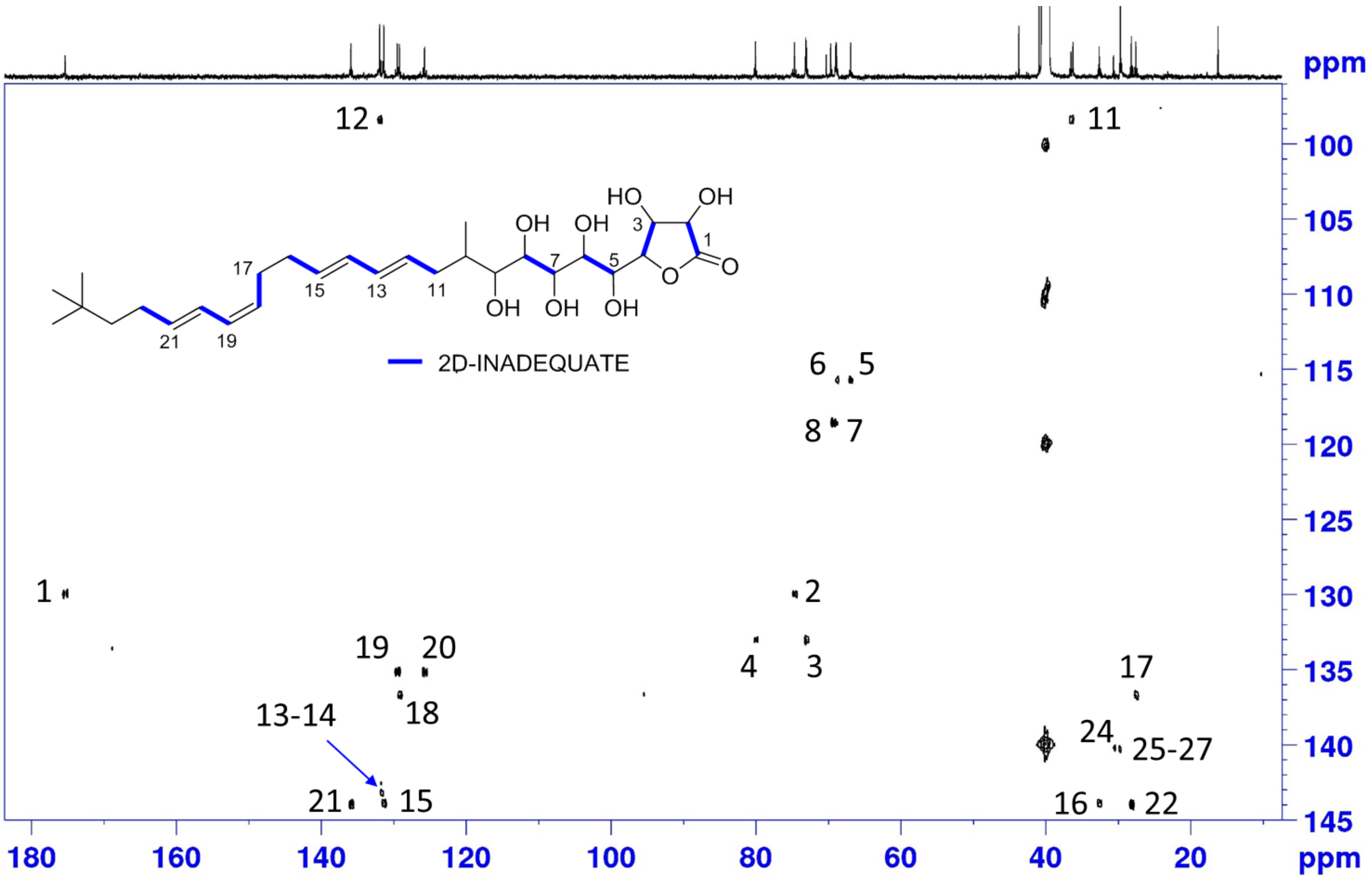

Figure S5: ^{13}C NMR spectrum of **1** labeled with [1-^{13}C]propionate (125 MHz, DMSO-d_6)

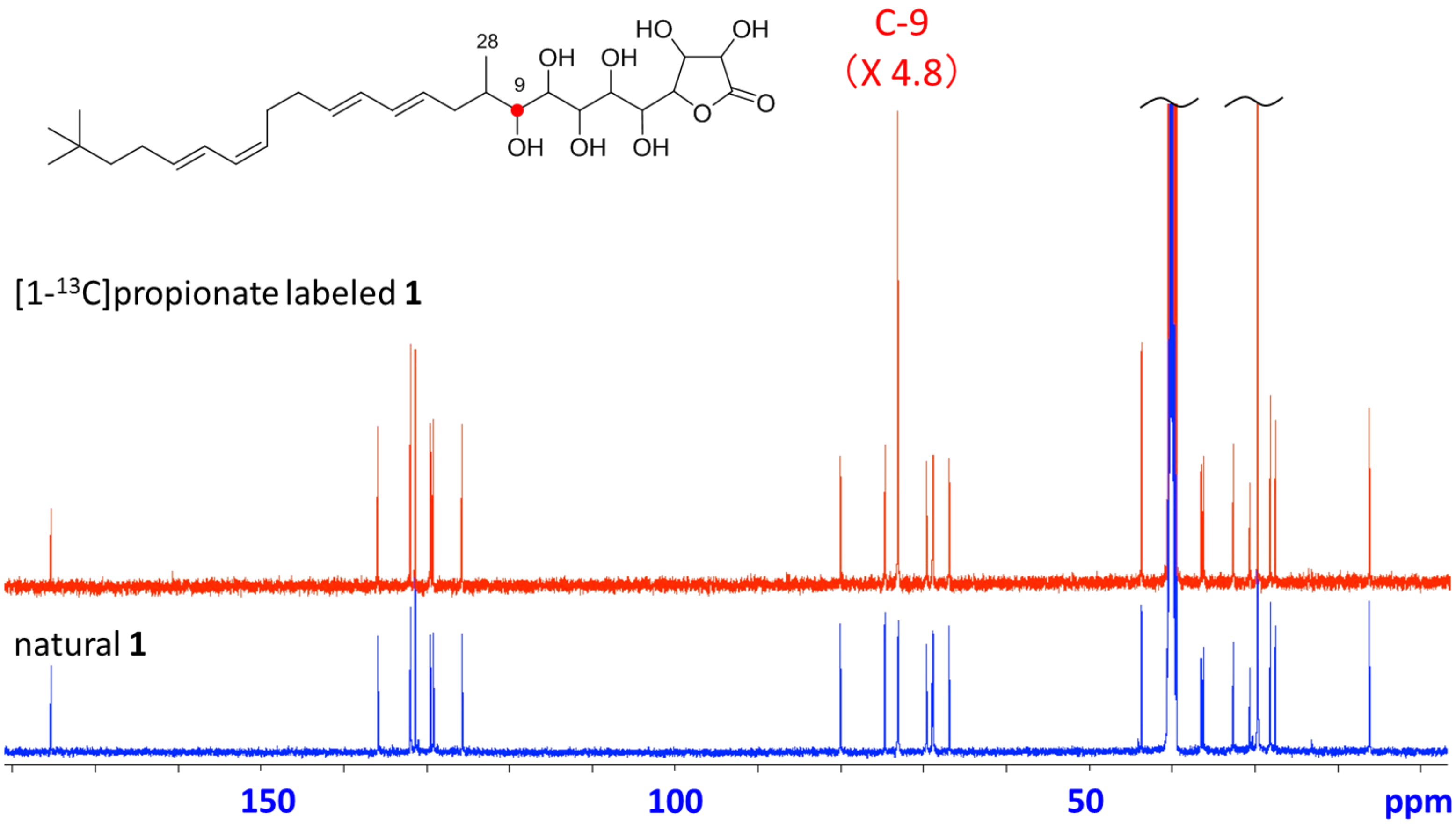

Figure S6: ^{13}C NMR spectrum of **1** labeled with L-[*methyl*-^{13}C]methionine (125 MHz, DMSO-d_6)

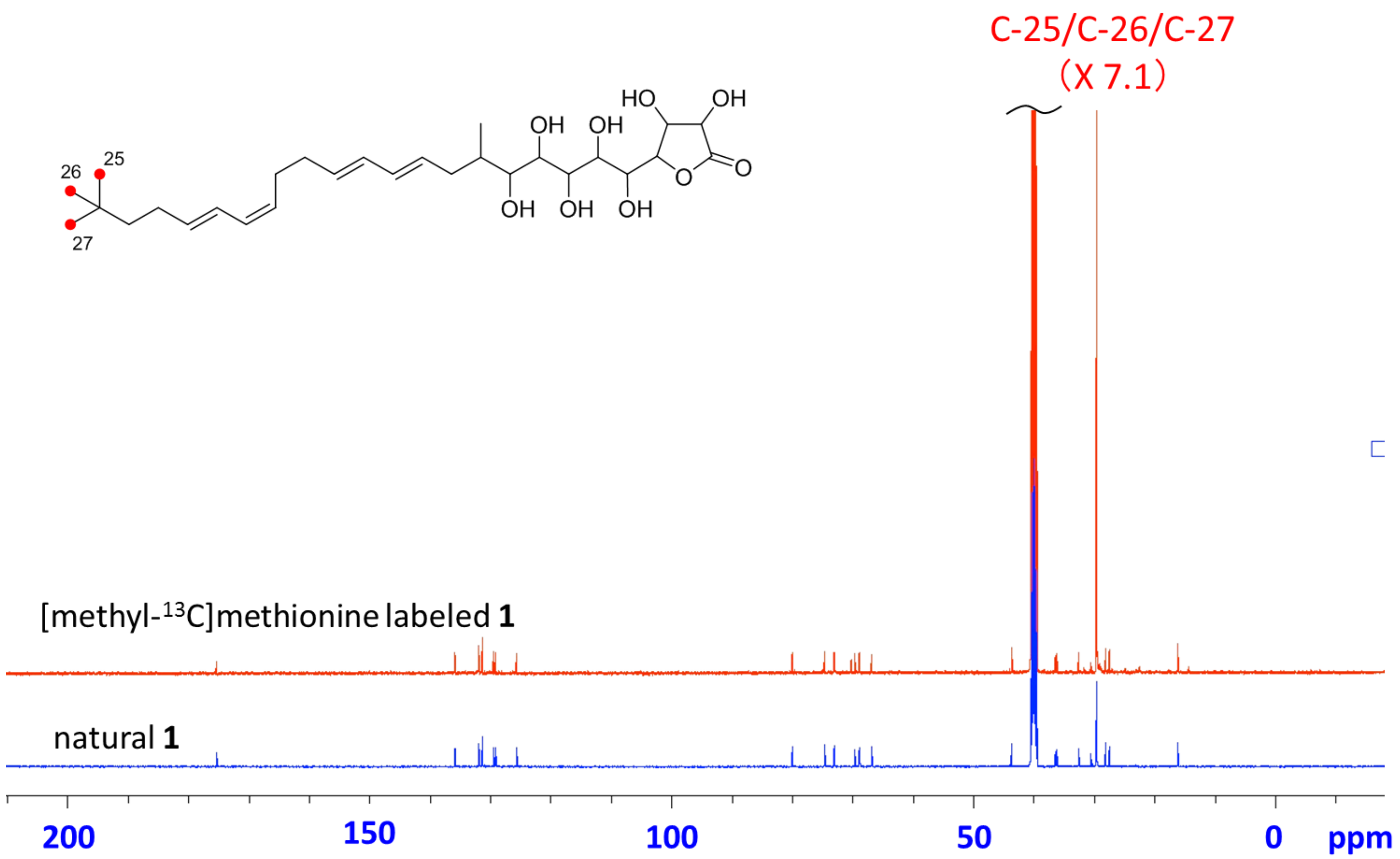

Figure S7: ^{1}H NMR spectrum of **1** (500 MHz, DMSO-d_6)

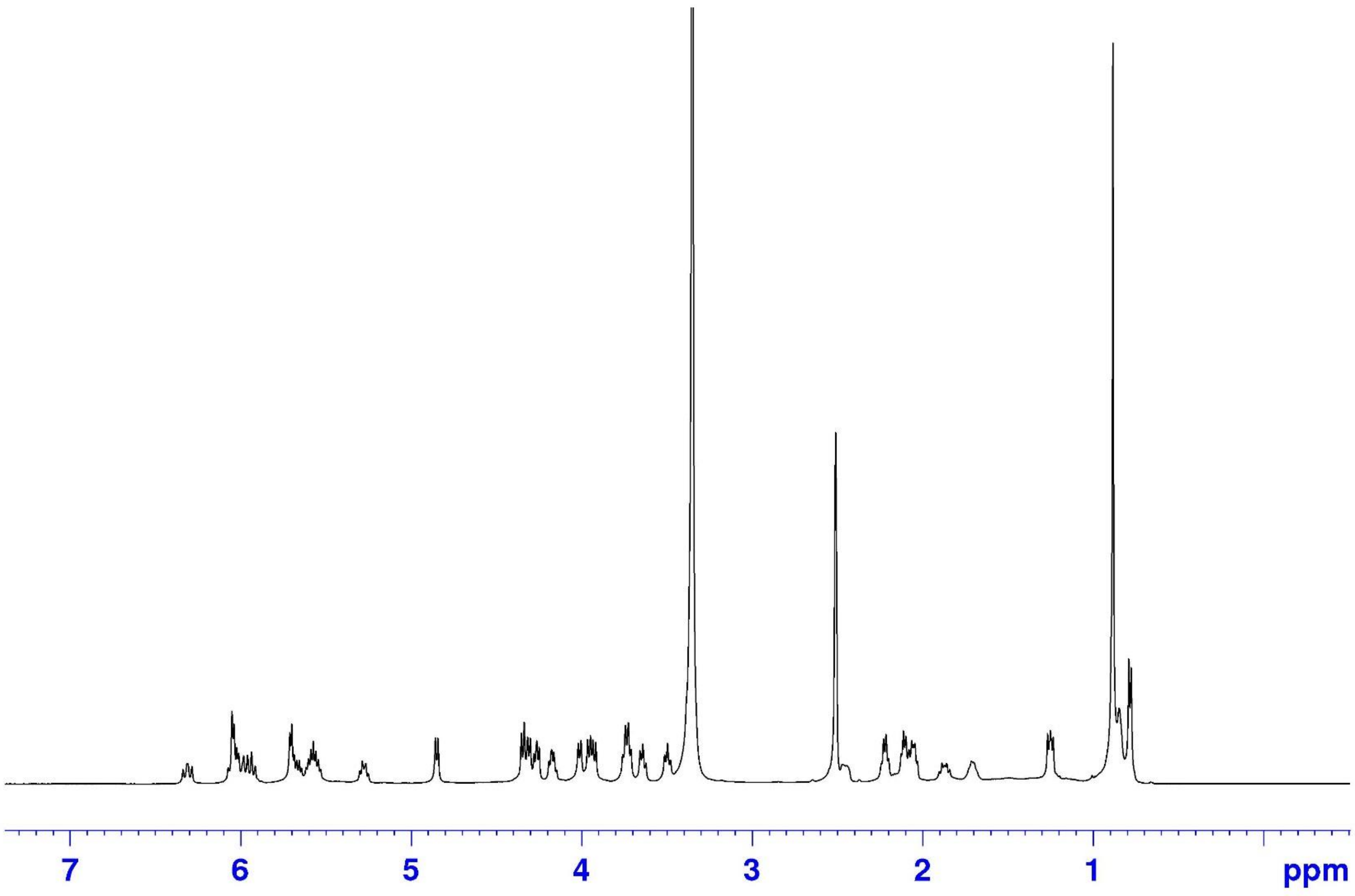

Figure S8: HRESITOFMS spectrum of **1**

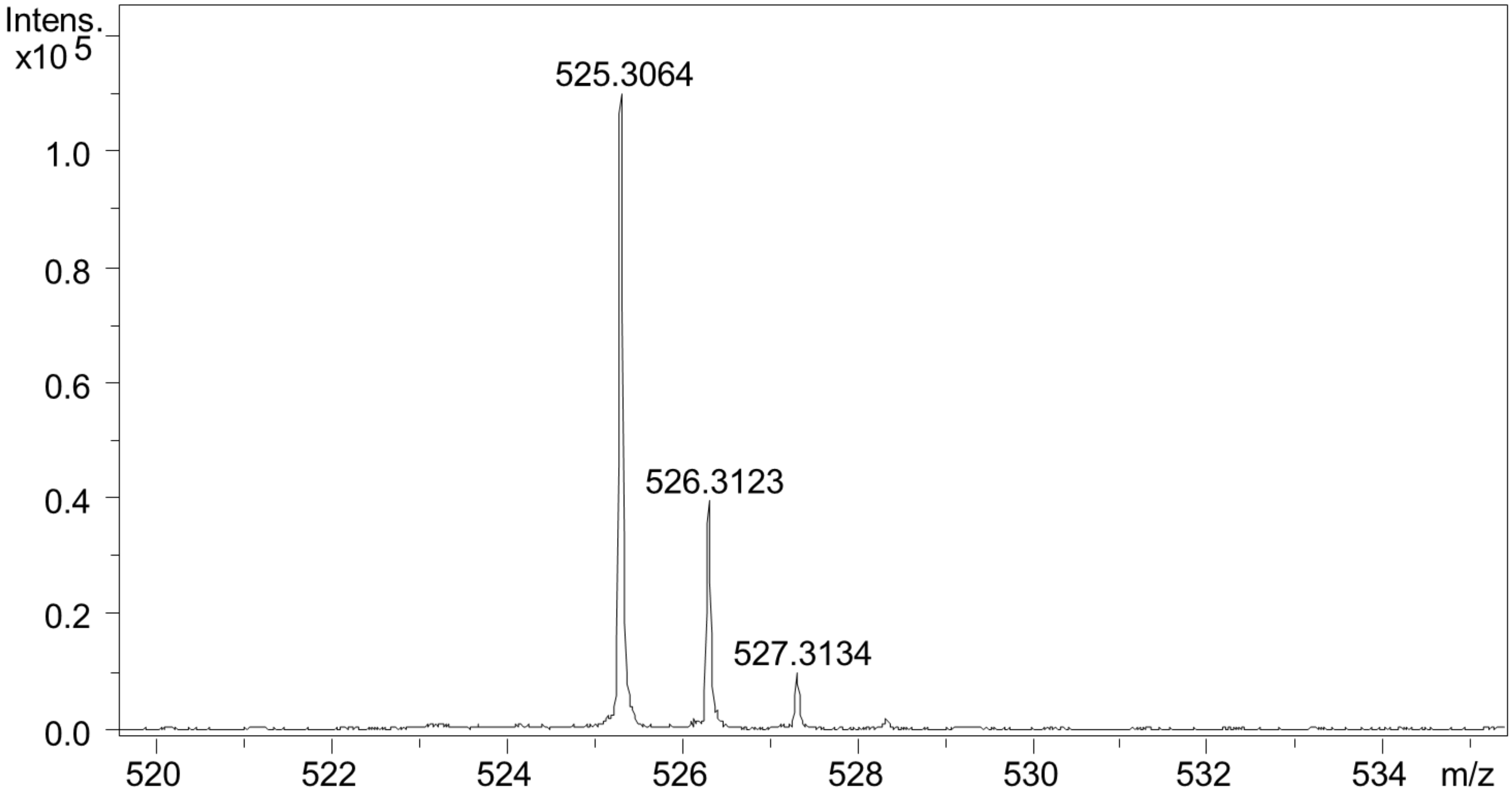

Supplement: File 1 — NMR spectra of 13C- and 2H-labeled 1. [file Beilstein_J_Org_Chem-13-441-s001.pdf]
